# The association between early corticosteroid use and the risk of secondary infections in hospitalized patients with COVID-19: a double-edged sword. Results from the international SCCM discovery viral infection and respiratory illness universal study (VIRUS) COVID-19 registry

**DOI:** 10.3389/fmed.2025.1466346

**Published:** 2025-02-14

**Authors:** Vikas Bansal, Nitesh K. Jain, Amos Lal, Anwar Khedr, Aysun Tekin, Abbas B. Jama, Noura Attallah, Esraa Hassan, Hisham Ahmed Mushtaq, Sara Robinson, Marjan Jahani Kondori, Thoyaja Koritala, Donna Lee Armaignac, Amy B. Christie, Umamaheswara Raju, Ashish Khanna, Rodrigo Cartin-Ceba, Devang K. Sanghavi, Abigail La Nou, Karen Boman, Vishakha Kumar, Allan J. Walkey, Juan Pablo Domecq, Rahul Kashyap, Syed Anjum Khan, the Society of Critical Care Medicine (SCCM) Discovery Viral Infection and Respiratory Illness Universal Study (VIRUS): COVID-19 Registry Investigator Group

**Affiliations:** ^1^Division of Nephrology and Critical Care Medicine, Department of Internal Medicine, Mayo Clinic, Rochester, MN, United States; ^2^Department of Critical Care Medicine, Mayo Clinic Health System, Mankato, MN, United States; ^3^Department of Critical Care Medicine, North East Georgia Health System, Gainesville, GA, United States; ^4^Department of Medicine, Division of Pulmonary and Critical Care Medicine, Multidisciplinary Epidemiology and Translational Research in Intensive Care Group (METRIC), Mayo Clinic, Rochester, MN, United States; ^5^Department of Family Medicine, Mayo Clinic Health System, Mankato, MN, United States; ^6^Center for Advanced Analytics, Baptist Health South Florida, Miami, FL, United States; ^7^Department of Trauma Critical Care, The Medical Center Navicent Health, Mercer University School of Medicine, Macon, GA, United States; ^8^Gandhi Medical College and Hospital, Hyderabad, India; ^9^Section on Critical Care Medicine, Department of Anesthesiology, Wake Forest University School of Medicine, Atrium Health Wake Forest Baptist Medical Center, Winston-Salem, NC, United States; ^10^Division of Critical Care Medicine, Department of Pulmonary Medicine, Mayo Clinic, Scottsdale, AZ, United States; ^11^Department of Critical Care Medicine, Mayo Clinic Florida, Jacksonville, FL, United States; ^12^Department of Critical Care Medicine, Mayo Clinic Health System, Eau Claire, WI, United States; ^13^Society of Critical Care Medicine, Mount Prospect, IL, United States; ^14^Pulmonary Center, Division of Pulmonary, Allergy, Critical Care and Sleep Medicine, Department of Medicine, Boston University School of Medicine, Boston, MA, United States; ^15^Division of Critical Care Medicine, Department of Anesthesiology and Perioperative Care, Mayo Clinic, Rochester, MN, United States; ^16^Department of Medicine and Medical, Research, WellSpan Health, York, PA, United States

**Keywords:** SARS-CoV-2, COVID-19, corticosteroids, secondary infections, hospitalized patients, risk factors, early steroid treatment outcomes, VIRUS COVID-19 registry

## Abstract

**Background:**

Corticosteroids improve survival in hospitalized COVID-19 patients needing supplemental oxygen. However, concern exists about increased risk of secondary infections. This study investigated the impact of early corticosteroids use on these infections.

**Methods:**

Data from the Society of Critical Care Medicine Discovery Viral Infection and Respiratory Illness Universal Study (VIRUS): COVID-19 registry were analyzed for adult patients, stratified by early corticosteroid use (within 48 h of admission). The primary outcome was documented secondary infections, including bacteremia, bacterial pneumonia, empyema, meningitis/encephalitis, septic shock, and ventilator-associated pneumonia. Univariate and multivariable logistic regression models were used to assess the association between early corticosteroids and these outcomes.

**Results:**

Among 17,092 eligible patients, with 13.5% developed at least one secondary bacterial infection during hospitalization. Patients receiving early corticosteroids were older (median 63 years) compared to those who did not (median 60 years), with a similar gender distribution (42.5% vs. 44.2% female). Unadjusted analysis revealed a higher risk for any secondary infection (OR 1.93, 95% CI 1.76–2.12). This association persisted for specific infections including bacteremia (OR 2.0, 95% CI 1.58–2.54), bacterial pneumonia (OR 1.5, 95% CI 1.27–1.77), and septic shock (OR 1.67, 95% CI 1.44–1.93). However, the effect on meningitis/encephalitis (OR 0.62, 95% CI 0.24–1.57) and ventilator-associated pneumonia (VAP; OR 1.08, 95% CI 0.75–1.57) was non-significant. Adjusted analysis maintained significance for any secondary infection (OR 1.15, 95% CI 1.02–1.29), bacteremia (OR 1.43, 95% CI 1.09–1.88), and infections with unknown sources (OR 1.63, 95% CI 1.31–2.02). Notably, the association weakened and became non-significant for bacterial pneumonia (OR 0.98, 95% CI 0.81–1.20) and septic shock (OR 0.94, 95% CI 0.79–1.11), while it became significant for meningitis/encephalitis (OR 0.26, 95% CI 0.08–0.82). VAP remained non-significant (OR 0.87, 95% CI 0.56–1.34).

**Conclusion:**

Early use of corticosteroids increased overall secondary infection risk in hospitalized COVID-19 patients, but the impact varied. Risk of bacteremia was notably increased, while the association with bacterial pneumonia and septic shock weakened after adjustment becoming non-significant and surprisingly reduced meningitis/encephalitis risk was noted suggesting the complexity of corticosteroid effects. Further research is needed to understand how corticosteroids influence specific secondary infections, and thereby optimize the treatment strategies.

## Introduction

COVID-19 pandemic caused by severe acute respiratory syndrome coronavirus 2 (SARS-CoV-2) has had far reaching social, political, and economic impact since it was first reported in November 2019. It has led to significant morbidity and mortality with a high rate of hospitalization. Multiple risk factors are involved in the disease prognosis, and patients infected with SARS-CoV-2 display a spectrum of clinical presentations that range from asymptomatic to life-threatening manifestation (1–7). For this multi-system disorder (8–12) different therapeutic strategies have been tested since the disease’s inception (13–18). The Mayo Clinic and Society of Critical Care (SCCM) Discovery Network created a global Viral Infection and Respiratory Illness Universal Study (VIRUS): COVID-19 registry for the collection of high-quality data in real-time (19) and for the study of COVID-19 treatments and related outcomes (20–22).

Historically pandemics such as influenza have had a high rate of mortality attributed to co-infections (23). Co-infections occur when a patient is infected with two or more pathogens (viruses, bacteria) at the same time (24). In contrast, the co-infection rate in COVID-19 patients was also noted, but much lower (around 3–8%) compared to historical influenza outbreaks (25, 26). However, occurrence of secondary infections, which develop after the initial COVID-19 infection (24), was noted to be above 40%, a rate much higher than the non-COVID-19 patients (27–33). A number of factors contributed to this secondary infection burden such as prolonged hospitalization, high device utilization rates such as central line and urinary catheter use, invasive mechanical ventilation, pandemic surge overwhelming healthcare capacity, high burn out rate, high comorbidity rate, and immunocompromised state of patients (33–36).

Corticosteroids have been used as therapeutic agents in viral pneumonia and Adult respiratory distress syndrome (ARDS) for many decades (37–39). However, their therapeutic benefit or harm was not well defined with contradictory results in ARDS studies and lack of benefit or even harm in Influenza pneumonia and Middle East Respiratory Syndrome (MERS) (40–42). Given this uncertainty and amidst the chaos of the COVID-19 pandemic, corticosteroids were shown to improve survival in patients with severe COVID-19 requiring supplemental oxygen and respiratory assist devices (40, 43–45). Indeed, the survival effect was more pronounced in critically ill patients needing invasive mechanical ventilation (40, 44, 46). However, the possibility of corticosteroids causing harm when used in patients with ARDS secondary to prolonged viral shedding, hyperglycemia secondary to use of corticosteroids, immunosuppression of the host and therefore increasing the risk of secondary infection remained unsettled (34, 36, 40, 47).

After the “Dexamethasone in Hospitalized Patients with COVID-19” (The RECOVERY collaborative group) (44), corticosteroids became a mainstay of treatment for treating hospitalized patients with severe COVID-19 requiring supplementation oxygen and respiratory assist devices. However, this landmark trial was not powered to detect the differences between the groups with regards to the secondary infection rate, a side effect profile of corticosteroid uses in COVID-19 patients (44). Many other randomized control trials which suspended recruitment post “Dexamethasone in Hospitalized Patients with COVID-19” reported a very small number of patients with secondary infection, which left this question unanswered (48, 49). Observational studies with their inherent confounding limitations reported conflicting results of corticosteroids being a risk for of secondary infections (27, 34–36, 42). There are several mechanisms on how corticosteroids might cause harm when used in patients with severe COVID-19. Prolonged viral shedding, steroid induced hyperglycemia, and immunosuppression are among the most accepted mechanism on how steroids might increase the risk of secondary infections, but still this important question remains unsettled (34, 36, 40, 47).

Considering the existing evidence of corticosteroid-induced immunosuppression and the high prevalence of secondary infections in hospitalized COVID-19 patients, this study aims to investigate the potential association between early corticosteroid use and secondary infection rates within a large, multicenter. Multinational SCCM Discovery VIRUS COVID-19 registry, a rich repository of demographics, treatment details, healthcare processes, hospital outcomes, and documented complications for hospitalized COVID-19 patients from 183 hospitals across 24 countries.

## Methods

### Overview of the VIRUS COVID-19 registry

Our analysis utilized data from the SCCM Discovery VIRUS COVID-19 registry, a comprehensive collection of clinical information for hospitalized patients of all ages confirmed to have SARS-CoV-2 infection (by PCR or similar methods) across 183 hospitals in 24 countries from March 1, 2020. The registry was established through the Society of Critical Care Medicine (SCCM) Discovery Network. Established in early 2020, the registry served as a consolidated international repository for ongoing COVID-19 related clinical research. Details of registry design, data management, process of resolution of qualitative and quantitative data related issues have been described elsewhere (20–22), and initial findings from the registry have been previously reported (2, 50).

The registry included patients from both intensive care units and general medical wards, encompassing a diverse spectrum of respiratory support needs. These needs ranged from supplemental oxygen to more advanced interventions like high-flow oxygen, noninvasive ventilation, invasive mechanical ventilation, proning, neuromuscular paralysis, and extracorporeal membrane oxygenation (ECMO). Patient were followed up until discharge or death, whichever occurred first. This registry received exempt status from human participant research review by the Mayo Clinic Institutional Review Board (IRB#: 20-002610) and is registered on Clinicaltrials.gov (NCT04323787). All participating investigative sites obtained local ethical approval and a data use agreement before data collection commenced. As stipulated in the approved protocol, informed consent was waived under Common Rule 45 CFR 46.116, and individual study sites signed a data use agreement to acquire permission for de-identified data extraction and entry into registry case report forms (CRFs). The case report forms were adapted from the World Health Organization templates (51) and modified for an ICU-specific context through rapid, iterative editing to balance feasibility, efficiency, and comprehensiveness, with input from multiple clinical specialties and adding pertinent data for clinical research.

### Study design and data source

This ancillary study utilizes data from the “SCCM Viral Infection and Respiratory Illness Universal Study (VIRUS) – COVID-19 registry.” We focused on adult patients aged 18 years and older, adhering to the Strengthening the Reporting of Observational Studies in Epidemiology (STROBE) guideline (52). Our primary exposure of interest was early administration of systemic corticosteroids within 48 h of hospital admission. Patients were categorized into two groups: those receiving early corticosteroids and those who did not. While complete corticosteroid treatment details (duration and type of corticosteroid formulation) were not captured, relevant elements like COVID-19 diagnosis confirmation, patient demographics, comorbid conditions, COVID-19 disease severity, early corticosteroid use, pre-hospital and in-hospital medications, hospital complications and pertinent clinical outcomes were collected from the VIRUS CRF.

### Exclusion criteria

Patients were excluded if:

1. Hospitalization was less than 48 h
2. They had a secondary or co-infection diagnosed within the first 48 h of hospitalization
3. Data was missing on medication use on days 0–2 and
4. Microbiology results or complications during hospitalization were missing.

### Infection classification

Admission diagnoses of bacteremia, bacterial pneumonia, meningitis/encephalitis, or positive microbiological findings within 48 h were classified as community-acquired infections and considered co-infections. Complications of bacteremia, empyema, endocarditis, lung abscess, septic shock, ventilator-associated pneumonia, and positive microbiological findings after 48 h were classified as hospital-acquired infections and considered as secondary infections.

### Primary outcome

The study’s primary focus was the occurrence of any documented secondary infection. This included infections like bacteremia, bacterial pneumonia, empyema, meningitis/encephalitis, septic shock, ventilator-associated pneumonia (VAP) and those with an unknown source.

### Statistical analysis

Continuous variables with normal distributions were summarized using means and standard deviations (SD). For non-normal continuous variables, medians, and interquartile ranges (IQRs) were reported, indicating the 25th and 75th percentiles. Categorical variables were presented as frequencies and percentages. Differences between groups for categorical variables were assessed using Pearson’s Chi-squared tests. To compare non-parametric continuous variables, a Kruskal-Wallis ANOVA was employed with subsequent Mann–Whitney U tests for pairwise comparisons. All analyses were conducted on available data, with the number of observations reported for each variable. Missing data was not imputed. We investigated the association between early in-hospital administration of systemic corticosteroids and these outcomes using both univariate and multivariable logistic regression models. We used existing literature and our univariate analysis to find certain patient characteristics and comorbidities associated with our outcomes of interest. These predictor variables were included into the multivariable model, after evaluating for collinearity. The multivariable logistic regression model adjusted for potential confounding factors, including age, gender, race, ethnicity, body mass index (BMI), ICU admission during index hospitalization, various comorbidities such as coronary artery disease (CAD), hypertension, heart failure, chronic obstructive pulmonary disease (COPD), asthma, chronic kidney disease, diabetes (DM), stroke/other neurological disorders, and dyslipidemia, the highest documented COVID-19 severity as per the WHO Ordinal scale during hospitalization, length of hospital stay, concurrent medication use (including tocilizumab, baricitinib), and antibacterials exposure within the first 48 h of admission. Finally, we compared the frequency of these outcomes between those who received early corticosteroids and those who did not. Diagnoses of these infections were based on the VIRUS registry records and were evaluated and documented as “yes” or “no” in the case report forms. All analyses were conducted using JMP® Software, version Pro 14 (SAS Institute Inc., Cary, NC, USA).

## Results

### Patient population and baseline characteristics

**Figure 1:**
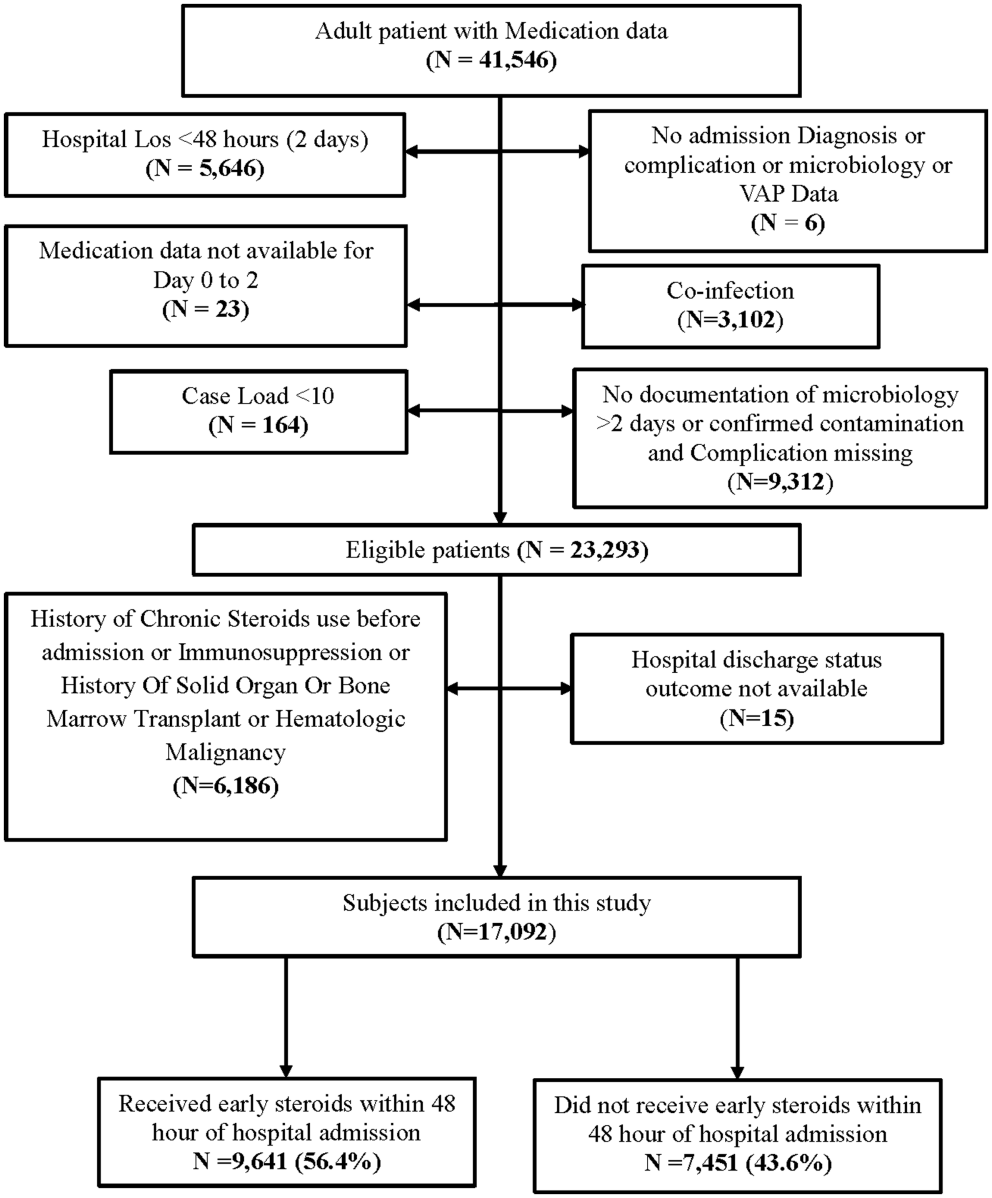
Consort diagram explaining patient inclusion scheme from the VIRUS registry.

A total of 17,092 patients met eligibility criteria between March 1, 2020, and March 14, 2023, and were included in the analysis (Figure 1). Of these, 9,641 received early inpatient systemic corticosteroids (Figure 1). Table 1 presents a comparison of baseline demographics between the early corticosteroid and control groups. Notably, the median age of patients receiving early corticosteroids was 63 years (IQR 51–75) with 42.5% female, compared to 60 years (IQR 45–74) with 44.2% female in the control group. Table 2 further details the patient characteristics of the index hospitalization based on early steroid use within 48 h of admission.

**Table 1:** Baseline demographics of patients in VIRUS COVID-19 registry by early steroid use within 48 hours of hospital admission.

Variable name	Received early steroids *N* = 9,641	Did not receive early steroids *N* = 7,451	Total *N* = 17,092	*p*-value
Age in year				
N^	9,641	7,451	17,092	<0.0001*
Median (IQR)	63 (51–75)	60 (45–74)	62 (49–75)	
Age group, n (%)				
N^	9,641	7,451	17,092	<0.0001*
18–40 years	1,172 (12.2)	1,473 (19.8)	2,645 (15.5)	
41–60 years	3,031 (31.4)	2,295 (30.8)	5,326 (31.2)	
>60 years	5,438 (56.4)	3,683 (49.4)	9,121 (53.4)	
Sex				
N^	9,641	7,451	17,092	0.0305*
Male, N (%)	5,543 (57.5)	4,160 (55.8)	9,703 (56.8)	
Female, N (%)	4,098 (42.5)	3,291 (44.2)	7,389 (43.2)	
Race, N (%)				
N^	9,641	7,451	17,092	<0.0001*
White Caucasian	6,849 (71)	4,210 (56.5)	11,059 (64.7)	
Black or African American	957 (9.9)	1,458 (19.6)	2,415 (14.1)	
Asian American	81 (0.8)	63 (0.8)	144 (0.8)	
Mixed Race	277 (2.9)	74 (1)	351 (2.1)	
Other	1,303 (13.5)	1,518 (20.4)	2,821 (16.5)	
Unknown	174 (1.8)	128 (1.7)	302 (1.8)	
Ethnicity, N (%)				
N^	9,641	7,451	17,092	<0.0001*
Not Hispanic or Latino	5,254 (54.5)	5,060 (67.9)	10,314 (60.3)	
Hispanic or Latino	3,534 (36.7)	1,399 (18.8)	4,933 (28.9)	
Unknown/Choose Not to Disclose	853 (8.8)	992 (13.3)	1845 (10.8)	
BMI in kg/m^2^, Median IQR				
N^	8,773	6,079	14,852	<0.0001*
Median (IQR)	30 (26–35.4)	28.5 (24.8–33.7)	29.4 (34.7–25.6)	
BMI Classification, N (%)				
N^	8,773	6,079	14,852	<0.0001*
Underweight (<18.5)	112 (1.3)	158 (2.6)	270 (1.8)	
Healthy weight (18.5 to < 25)	1,491 (17)	1,424 (23.4)	2,915 (19.6)	
Overweight (25 to < 30)	2,772 (31.6)	1,961 (32.3)	4,733 (31.9)	
Class 1 Obesity (30 to < 35)	2,070 (23.6)	1,273 (20.9)	3,343 (22.5)	
Class 2 Obesity (35 to < 40)	1,188 (13.5)	638 (10.5)	1826 (12.3)	
Class 3 Obesity (≥40)	1,140 (13)	625 (10.3)	1765 (11.9)	
Comorbidities, n (%)				
N^	9,641	7,451	17,092	
Obesity	4,818 (50.0)	2,747 (36.9)	7,565 (44.3)	<0.0001*
Coronary Artery Disease	771 (8.0)	831 (11.2)	1,602 (9.4)	<0.0001*
Hypertension	5,773 (59.9)	4,296 (57.7)	10,069 (58.9)	0.0035*
Congestive Heart Failure	1,077 (11.2)	748 (10.0)	1825 (10.7)	0.0177*
Chronic Obstructive Pulmonary Disease	1,234 (12.8)	522 (7.0)	1756 (10.3)	<0.0001*
Asthma	461 (4.8)	385 (5.2)	846 (5.0)	0.2553
Chronic Kidney Disease^$^	1,281 (13.3)	1,100 (14.8)	2,381 (13.9)	0.0061*
Diabetes Mellitus	3,280 (34.0)	2,519 (33.8)	5,799 (33.9)	0.7818
Stroke Or Other Neurological Disorders	620 (6.4)	565 (7.6)	1,185 (6.9)	0.0035*
Dyslipidemia	1719 (17.8)	1,223 (16.4)	2,942 (17.2)	0.0151*
Social History				
N^	9,641	7,451	17,092	
Current Smoker	439 (4.6)	480 (6.4)	919 (5.4)	<0.0001*
Former Smoker	1,334 (13.8)	1,163 (15.6)	2,497 (14.6)	0.0012*

^Total number of responses available for analysis for respected variables after excluding missing data. ^$^Includes chronic kidney disease stages 1–5.

BMI, Body mass index; IQR, Interquartile range. * *p*-value <0.05.

**Table 2:** Patient characteristics of the index hospitalization in VIRUS COVID-19 registry by early steroid use within 48 hours of hospital admission.

Variable name	Received early steroids *N* = 9,641	Did not receive early steroids *N* = 7,451	Total *N* = 17,092	*p*-value
Admission diagnosis				
N^	9,641	7,451	17,092	
Acute Hypoxic Respiratory Failure (Non-ARDS), n (%)	4,929 (51.1)	2,218 (29.8)	7,147 (41.8)	<0.0001*
Acute Liver Injury, n (%)	21 (0.2)	61 (0.8)	82 (0.5)	<0.0001*
Acute myocardial infarction, n (%)	42 (0.4)	54 (0.7)	96 (0.6)	0.0132*
Acute kidney injury, n (%)	392 (4.1)	506 (6.8)	898 (5.3)	<0.0001*
Acute respiratory distress syndrome (ARDS), n (%)	315 (3.3)	221 (3.0)	536 (3.1)	0.2688
Congestive heart failure, n (%)	100 (1.0)	151 (2.0)	251 (1.5)	<0.0001*
Diabetic Ketoacidosis (DKA), n (%)	27 (0.3)	50 (0.7)	77 (0.5)	0.0002*
Gastrointestinal Hemorrhage, n (%)	29 (0.3)	44 (0.6)	73 (0.4)	0.0044*
Stroke	70 (0.7)	131 (1.8)	201 (1.2)	<0.0001*
Concurrent Hospital Medications, N (%)				
N^	9,641	7,451	17,092	
Tocilizumab, n (%)	785 (8.1)	195 (2.6)	980 (5.7)	<0.0001*
Baricitinib, n (%)	451 (4.7)	25 (0.3)	476 (2.8)	<0.0001*
Antibacterials, n (%)	5,872 (60.9)	3,688 (49.5)	9,560 (55.9)	<0.0001*
Highest COVID-19 Disease severity as per WHO Ordinal Scale				
N^	9,600	7,332	16,932	<0.0001*
COVID-19 Disease severity Moderate, n (%), (WHO Ordinal Scale 4 & 5)	4,066 (42.4)	4,282 (58.4)	8,348 (49.3)	
COVID-19 Disease severity Severe, n (%), (WHO Ordinal Scale 6,7,8 & 9)	5,534 (57.6)	3,050 (41.6)	8,584 (50.7)	
IMV utilization				
N^	9,641	7,451	17,092	<0.0001*
IMV utilization, n (%)	2027 (21.0)	993 (13.3)	3,020 (17.7)	
IMV Days				
N^	1,660	895	2,555	0.0260*
Median (IQR)	7 (3.4–12.2)	6.2 (3–11.2)	7 (3.2–12)	
NIMV Utilization				
N^	9,641	7,451	17,092	<0.0001*
NIMV utilization, n (%)	2,467 (25.6)	655 (8.8)	3,122 (18.3)	
NIMV Days				
N^	1,361	477	1838	0.0246*
Median (IQR)	1.7 (0.6–4)	2 (0.9–4.6)	1.8 (0.7–4)	
HFNC Utilization				
N^	9,641	7,451	17,092	<0.0001*
HFNC utilization, n (%)	3,542 (36.7)	885 (11.9)	4,427 (25.9)	
HFNC Duration				
N^	2081	654	2,735	0.0398*
Median (IQR)	3.6 (1.6–6.5)	3.1 (1.0–6.0)	3.5 (1.5–6.4)	
ECMO Utilization				
N^	9,641	7,451	17,092	0.8026
ECMO Utilization, n (%)	38 (0.4)	27 (0.4)	65 (0.4)	
ECMO Duration				
N^	31	16	47	0.8221
Median (IQR)	8.0 (5.6–14)	9.5 (3.5–14.7)	9 (5.0–14.1)	
RRT Utilization				
N^	1,127	826	1953	0.0689
RRT Utilization, n (%)	179 (15.9)	158 (19.1)	337 (17.3)	
RRT Duration				
N^	176	156	332	0.7689
Median (IQR)	4.0 (2.0–8.0)	4.4 (2.4–8)	4 (2.1–8)	
ICU Admission Rate				
N^	9,585	7,304	16,889	<0.0001*
ICU Admission Rate, n (%)	3,825 (39.9)	2,351 (32.2)	6,176 (36.6)	
ICU Length of stay, Median IQR				
N^	3,557	2,265	5,822	<0.0001*
Median (IQR)	6.4 (3.0–11.2)	5.6 (2.4–10.3)	6 (3.0–11)	
ICU Mortality Rate				
N^	3,565	2,328	5,893	0.9272
ICU Mortality, n (%)	918 (25.8)	602 (25.9)	1,520 (25.8)	
Hospital Length of stay, Median IQR				
N^	9,641	7,451	17,092	<0.0001*
Median (IQR)	7.6 (4.9–13)	6.6 (4–11.7)	7.0 (4.4–12.3)	
Hospital Length of stay by Group, n (%)				
N^	9,641	7,451	17,092	<0.0001*
≤ 7 days	4,508 (46.8)	4,092 (54.9)	8,600 (50.3)	
> 7 to ≤ 14 days	3,028 (31.4)	2086 (28.0)	5,114 (29.9)	
> 14 days to 30 days	2,105 (21.8)	1,273 (17.1)	3,378 (19.8)	
Hospital Mortality Rate				
N^	9,641	7,451	17,092	<0.0001*
Hospital Mortality, n (%)	1734 (18.0)	813 (10.9)	2,547 (14.9)	
Hospital Discharge Location for Alive status, N (%)				
N^	7,897	6,597	14,494	<0.0001*
Home, without assistance	4,867 (61.6)	4,317 (65.4)	9,184 (63.4)	
Home, with home health	1796 (22.7)	1,116 (16.9)	2,912 (20.1)	
Subacute rehabilitation	279 (3.5)	248 (3.8)	527 (3.6)	
Long-term care facility	577 (7.3)	535 (8.1)	1,112 (7.7)	
Hospice	161 (2)	120 (1.8)	281 (1.9)	
Other hospital (overflow)	79 (1)	69 (1.1)	148 (1)	
Other	138 (1.8)	192 (2.9)	330 (2.3)	
Any documented Secondary infection including Ventilator-associated pneumonia (VAP)				
N^	9,641	7,451	17,092	<0.0001*
Any documented Secondary infection	1,603 (16.6)	697 (9.4)	2,300 (13.5)	
Breakdown of Secondary infection as hospital complication				
Bacteremia, n (%)	243 (2.5)	95 (1.3)	338 (2.0)	<0.0001*
Bacterial Pneumonia, n (%)	429 (4.5)	224 (3.0)	653 (3.8)	<0.0001*
Empyema, n (%)	5 (0.05)	0 (0.0)	5 (0.03)	0.0728
Meningitis/Encephalitis	8 (0.08)	10 (0.1)	18 (0.1)	0.3466
Septic Shock	600 (6.2)	285 (3.8)	885 (5.2)	<0.0001*
Secondary infection unknown source	516 (5.4)	152 (2.0)	668 (3.9)	<0.0001*
Documented Ventilator-associated pneumonia (VAP)				
N^	937	775	1712	0.7064
VAP, n (%)	69 (7.4)	53 (6.8)	122 (7.1)	

^Total number of responses available for analysis for respected variables after excluding missing data.

ARDS, Acute respiratory distress syndrome; ECMO, Extracorporeal membrane oxygenation; HFNC, High Flow Nasal Cannula; ICU, Intensive Care Unit; IMV, Invasive Mechanical Ventilation; NIMV, Non-invasive Mechanical Ventilation; RRT, Renal Replacement Therapy; VAP, Ventilator-associated pneumonia. * *p*-value <0.05.

### Secondary infection rates and association with corticosteroids

Overall, 13.5% of patients developed at least one of the studied secondary bacterial infections during their hospitalization. Unadjusted analysis (Table 3) revealed that early corticosteroid administration significantly increased the odds of any secondary infection (OR 1.93, 95% CI 1.76–2.12; *p*-value <0.001). This association remained significant for specific infections like bacteremia (OR 2.0, 95% CI 1.58–2.54; *p*-value <0.001), bacterial pneumonia (OR 1.5, 95% CI 1.27–1.77; *p*-value <0.001), septic shock (OR 1.67, 95% CI 1.44–1.93; *p*-value <0.001), and secondary infection from unknown source (OR 2.72, 95% CI 2.26–3.26, *p*-value <0.001).

**Table 3:** Unadjusted and adjusted risk of secondary infections with early steroid use within 48 hours of hospital admission in hospitalized COVID-19 patients.

	Unadjusted		Adjusted	
Outcome variables	OR (95% CI)	*p*-value	OR (95% CI)	*p*-value
Any Secondary infection	1.93 (1.76–2.12)	<0.001*	1.15 (1.02–1.29)	0.023*
Breakdown of Secondary infection as hospital complication				
Bacteremia	2.0 (1.58–2.54)	<0.001*	1.43 (1.09–1.88)	0.010*
Bacterial pneumonia	1.5 (1.27–1.77)	<0.001*	0.98 (0.81–1.2)	0.870
Empyema	Unstable due to low numbers of documented Empyema Complication			
Meningitis/encephalitis	0.62 (0.24–1.57)	0.347	0.26 (0.08–0.82)	0.021*
Septic Shock	1.67 (1.44–1.93)	<0.001*	0.94 (0.79–1.11)	0.468
VAP	1.08 (0.75–1.57)	0.706	0.87 (0.56–1.34)	0.527
Secondary infection unknown source	2.72 (2.26–3.26)	<0.001*	1.63 (1.31–2.02)	<0.001*

CI, Confidence Interval; MD, Mean difference; SE, Standard error; ICU, Intensive Care Unit.

Adjusted for age, gender, race, ethnicity, BMI, ICU admission on index hospitalization, highest documented COVID-19 severity as per the WHO Ordinal scale during hospitalization, Hospital Length of stay and co-current medication (tocilizumab and Baricitinib), antibacterial exposure, and comorbidities, such as Chronic Kidney Disease, Chronic Pulmonary Disease, Congestive Heart Failure, Coronary Artery Disease, Diabetes (DM), Dyslipidemia/Hyperlipidemia, Hypertension, and Stroke Or Other Neurological Disorders.

After adjusting for potential confounding factors, the association between early corticosteroid use and secondary infections persisted for some but not all outcomes. The adjusted odds of developing any secondary infection (OR 1.15, 95% CI 1.02–1.29; *p*-value = 0.023), bacteremia (OR 1.43, 95% CI 1.09–1.88; *p*-value = 0.010), meningitis/encephalitis (OR 0.26, 95% CI 0.08–0.82; *p*-value = 0.021), and secondary infection from unknown source (OR 1.63, 95% CI 1.31–2.02, *p*-value <0.001) remained statistically significant. However, the association with bacterial pneumonia and septic shock did not reach statistical significance in the adjusted model (OR 0.94, 95% CI 0.79–1.11, *p*-value = 0.468).

## Discussion

The administration of corticosteroids early in the course of hospitalization for COVID-19 significantly increased the overall risk of secondary infections. However, the effects on specific infection types were heterogeneous. Bacteremia risk was substantially elevated, while the associations with bacterial pneumonia and septic shock became less evident after controlling for potential confounding factors. Notably, meningitis/encephalitis exhibited a surprising trend toward decreased risk.

The overall rate of secondary infection in our study was 13.5% with the early corticosteroid group having a rate of 16.6% and non-corticosteroid group a rate of 9.4%. This secondary rate of infection in our study was consistent with other reported studies in literature elsewhere (53, 54). The rate of secondary infections in COVID-19 has been found to be higher than in the pre COVID-19 time period (34, 53, 54). As noted previously, this has been attributed to a number of causes unique to the pandemic such as excessive use of antibiotics, lax infection control measures during pandemic, prolonged hospitalization especially in critically ill patients associated with increased device utilization rates, and ventilator associated pneumonia amongst others (34, 53, 54).

Our study demonstrated increased secondary infection rate, bacteremia, and secondary infection from an after unknown source after adjustment, whereas no difference was noted in bacterial pneumonia, VAP, and septic shock. This increased rate of infection is remarkably similar to other reported retrospective large series such as the ESICM UNITE-COVID study (34). Furthermore, a study on mechanically ventilated patients also reported an increased risk of bacterial pneumonia and fungal infections with dexamethasone, emphasizing superinfections in critically ill patients (55). Similarly, another study focused on ICU patients found that prolonged corticosteroid use significantly raised the risk of bloodstream infections, consistent with our observation of increased bacteremia risk (56). It is noteworthy that the early steroid group had an increased incidence of acute hypoxic respiratory failure at admission and a higher COVID-19 disease severity scale. Interestingly, the early steroid group had a higher incidence of severe illness at admission, indicated by increased acute hypoxic respiratory failure scores and higher COVID-19 disease severity scales. This likely explains the association with a greater use and duration of respiratory support devices, including invasive mechanical ventilation (IMV) in the early steroid group. Additionally, the early steroid group had a higher ICU admission rate, longer ICU and hospital lengths of stay, and increased hospital mortality, but not ICU mortality. It is important to note that our study covered a period of nearly 3 years, encompassing both the pre and post-RECOVERY trial period. The RECOVERY trial (44) established the survival benefits of corticosteroids in COVID-19, likely leading to the widespread adoption of dexamethasone during the latter part of the study timeframe. This shift in standard care practices might explain why most patients requiring respiratory support received dexamethasone in the later stages of our study. However, the widespread adoption of corticosteroids as standard care after this trial makes it difficult to disentangle the independent effect of corticosteroids on infection rates. Similar to the ESICM UNITE-COVID study (34), we did not observe an overall ICU mortality benefit, but a trend toward increased mortality in the entire hospitalized population receiving early steroids. While our study was not designed to assess mortality definitively, this could be due to the higher acuity of illness in the group receiving early corticosteroids.

However, our results differ from other studies, such as the Mount Sinai COVID Informatics Center (MSCIC) analysis, which reported a lower coinfection rate in patients receiving corticosteroids (57). The MSCIC data consisted of more than 4,000 patients but also was reported early in the pandemic (54). Similarly, a study of critically ill patients found no significant association between corticosteroid use and secondary infections, contrasting with our findings of increased overall risk (58). This study focused on critically ill ICU patients with severe COVID-19, who were receiving intensive care and mechanical ventilation. In contrast to both studies our study consists of patients both from the general medical ward and ICU, spread out over a three-year period, during which time care for COVID-19 patients gradually became more standardized with hospitals only experiencing periods of intermittent surge. Thus, our results are more generalizable, more reflective of and applicable to real world with less bias.

Our study explores the link between corticosteroids and secondary infections in COVID-19 patients. While our data hints at fewer cases of meningitis/encephalitis with steroid use, the small group (18 people) limits firm conclusions. COVID-19 itself can cause this complication, as shown by a study of 32 cases by Huo et al. (59). The same study mentions individual cases where meningitis/encephalitis patients improved after getting steroids. This suggests that the benefit we observed might not be entirely due to steroids alone. It highlights the need to explore how these complications arise and how steroids influence their development. In hospitalized COVID-19 patients, meningitis and encephalitis are less often linked to secondary bacterial infections. Instead, they are usually caused by immune-driven inflammation in the brain or the virus directly invading the nervous system (59). Corticosteroids like dexamethasone help reduce cytokine storms and brain inflammation, which might explain the lower risk of these complications with steroid use (60). However, our preliminary finding of decreased incidence needs further research with larger groups and stronger methods to definitively assess this, confirm or refute our findings, uncover the underlying mechanisms, and thereby guide optimal clinical management.

Our study investigates the complex relationship between early corticosteroid use and secondary infections in COVID-19, offering several key strengths. This large-scale, international registry study (20–22) encompasses data from diverse hospitals across the globe, providing a robust and generalizable perspective on this critical relationship. Focusing on real-world data from actual clinical settings directly translates our findings to everyday patient care, enhancing their relevance and applicability. Furthermore, the comprehensive analysis of a wide range of secondary infections provides a clear picture of the potential risks associated with early corticosteroids. Importantly, we adjusted for various potential confounders, strengthening the validity and reliability of our observed associations. Finally, the consistency of our results with other large studies further reinforces the credibility and reproducibility of this association (53).

However, it is crucial to acknowledge the study’s limitations, which necessitate further research. While this observational study offers valuable real-world insights, it cannot definitively prove causality, and the retrospective design may limit the accuracy and completeness of data, especially when it comes to secondary infections like VAP and bacteremia. Additionally, variations in treatment regimes, patient populations across hospitals, and viral strains during different surge periods could have influenced our findings, which could not be adjusted in our study. Notably, different corticosteroid formulations, doses, and durations were employed based on local practices, and although this is unlikely to significantly impact our secondary infection results given the consistent effect observed in previous studies (43, 61), it warrants further investigation. Similarly, the determination of secondary infections relied on investigator judgment and reporting across multiple sites, introducing potential bias due to non-standardization. This is unavoidable given the geographic spread of hospitals and the multitude of investigators involved. The analysis of microbiological data also faces limitations. Firstly, data collection depends on what researchers recorded, potentially introducing bias, particularly for free-text information like antimicrobial use. Secondly, we cannot directly link microbiological findings to clinical outcomes. Thirdly, excluding patients with missing data may overestimate infection rates, as missing data is more likely for negative cultures. Additionally, for bloodstream and urinary tract infections, it is difficult to distinguish catheter-related infections. Finally, evaluating multi-bacterial culture positivity and microorganism susceptibility presents challenges. Despite these limitations, the consistency of our findings with other large studies is reassuring (34).

### Future research and recommendations

While our study has provided valuable information on how early corticosteroid use, COVID-19 itself, and secondary infections are linked, there are still unanswered questions. We need to understand better how exactly early steroids affect the risk of secondary infections; especially why different types of infections are impacted differently. Additionally, figuring out the exact role of COVID-19 versus corticosteroids in causing secondary infections requires studies that look closely at the types of pathogens involved and potentially treatments aimed at specific parts of the immune system. Investigating specific pathogens and their susceptibility to corticosteroids could provide more refined understanding of the risk profile. To improve patient outcomes, future research should explore the possibility of personalized corticosteroid plans based on individual risk factors for secondary infections and tailored to different types of COVID-19. Additionally, investigating additional treatments that could reduce the increased risk of secondary infections associated with early corticosteroids could be helpful in clinical practice. By addressing these remaining questions thorough research, we can get a clear picture of the complex relationship between COVID-19, corticosteroids, and secondary infections, ultimately leading to better treatment options and improved patient outcomes.

## Conclusion

Our study suggests that early in-hospital corticosteroids use significantly increased the risk of any secondary infection in patients with COVID-19, but their effect on specific infections varied significantly. While bacteremia risk substantially increased, associations with bacterial pneumonia and septic shock was weakened upon adjusting for confounding factors. Notably, meningitis/encephalitis showed a surprising decreased incidence, highlighting a critical knowledge gap in the existing data. This is particularly concerning considering that existing data from trials like the RECOVERY collaboration group and others did not report on secondary infections. This emphasizes the urgent need for careful consideration of both potential benefits and risks when using corticosteroids in this setting. Future randomized controlled trials should explicitly address the potential risk of secondary infections as an outcome to fully assess the risk–benefit profile of corticosteroid therapy so as to guide optimal clinical management.

## Data Availability

The original contributions presented in the study are included in the article/Supplementary material, further inquiries can be directed to the corresponding author.

## From the society of critical care medicine (SCCM) discovery viral infection and respiratory illness universal study (VIRUS): COVID-19 registry investigator group

SCCM Discovery VIRUS investigators collaborative co-author list is in the Acknowledgements section. The journal is requested to PubMed index the list of VIRUS: COVID-19 Registry Investigator Group as collaborative co-authors submitted along with this manuscript.
