# Supplementary material for: The association between early corticosteroid use and the risk of secondary infections in hospitalized patients with COVID-19: a double-edged sword. Results from the international SCCM discovery viral infection and respiratory illness universal study (VIRUS) COVID-19 registry
Source: Front Med (Lausanne). 2025 Feb 14;12:1466346. doi: 10.3389/fmed.2025.1466346 (PMC11868930; doi:10.3389/fmed.2025.1466346)
Supplement: Supplementary file 1 [file Supplementary_file_1.docx]

**Appendix 1:**

**Collaborative Co-Author List: The Association Between Early Corticosteroid Use and the Risk of Secondary Infections in Hospitalized Patients with COVID-19: A Double-Edged Sword. Results from the International SCCM Discovery Viral Infection and Respiratory Illness Universal Study (VIRUS) COVID-19 Registry**

**Belgium**

The Brugmann University hospital, Bruxelles: Philippe Clevenbergh

Centre Hospitalier Jolimont: Jean-Baptiste Mesland, Pierre Henin,  Hélène Petre, Isabelle Buelens, Anne-Catherine Gerard

**Bolivia**

Clinica Los Olivos: Rolando Claure-Del Granado, Jose A. Mercado, Esdenka Vega-Terrazas, Maria F. Iturricha-Caceres

**Canada**

Mackenzie Health, Richmond Hill: Ruben Garza, Eric Chu, Victoria Chan

**Columbia**

Clinica Medical SAS: Oscar Y Gavidia, Felipe Pachon

**Egypt**

Helwan University: Mohamed El Kassas, Ahmed Tawheed

**Greece**

ICU University Hospital of Ioannina: Dimitrios Kantas, Vasileios Koulouras

**Honduras**

Honduras Medical Center: Gabina María Reyes Guillen, Helin Archaga Soto, Ana Karen Vallecillo Lizardo

St. Mary Medical Center, Langhorne: Umang Patel, Jordesha Hodge, KrunalKumar Patel, Shivani Dalal, Himanshu Kavani, Sam Joseph

CEMESA Hospital: Estela Pineda

**India**

Gandhi Medical College and Hospital, Hyderabad: Umamaheswara Raju, Janaki Manduva, Naresh Kolakani, Shreeja Sripathi, Sheetal Chaitanya

Panimalar Medical College Hospital & Research Institute: Surapaneni Krishna Mohan, Ekambaram Jyothisree

BSES MG Hospital, Mumbai: Girish Vadgaonkar, Rekha Ediga, Shilpa Basety, Shwetha Dammareddy, Phani Sreeharsha Kasumalla

Om Superspeciality Hospital, Raipur: Kamlesh Kumar Agrawal, Vijendra Baghel, Kirti Kumar Patel

Medicover Hospitals: Sridhar Papani, Mahesh Kamuram

Maulana Azad Medical College and Lok Nayak hospital: Mradul Kumar Daga, Munisha Agarwal , Ishan Rohtagi

Jawaharlal Institute of Postgraduate Medical Education and Research, Pondicherry: Anusha Cherian, Sreejith Parameswaran , Magesh Parthiban, Menu Priya A.

**Japan**

Sapporo City General Hospital: Yuki Itagaki, Akira Kodate, Reina Suzuki, Akira Kodate,Yuki Takahashi, Koyo Moriki

Hiroshima University: Michihito Kyo

**Kuwait**

Al-Amiri and Jaber Al-Ahmed Hospitals, Kuwait Extracorporeal Life Support Program: Abdulrahman Al-Fares

**Mexico**

Hospital Universitario, Universidad Autonoma de Nuevo León: Rene Rodriguez-Gutierrez, Jose Gerardo Gonzalez-Gonzalez, Alejandro Salcido-Montenegro, Adrian Camacho-Ortiz

**Nigeria**

Aminu Kano Teaching Hospital/Bayero University, Kano: Fatimah Hassan-Hanga, Hadiza Galadanci, Abubakar Shehu Gezawa, Halima M. S. Kabara,Taiwo Gboluwaga Amole, Halima Kabir, Dalha Gwarzo Haliru, Abdullahi S Ibrahim

**Pakistan**

Dow University Hospital: Muhammad Sohaib Asghar, Mashaal Syed, Syed Anosh Ali Naqvi, Farah Yasmin, and Iftekhar Ahmed

Civil Hospital, Karachi: Muhammad Tanveer Alam, Arjan Kumar, Imran Sarwar Shaikh, Hafsa Nazir Jatoi, Muhammad Rehan, and Rohan Kumar Ochani

**Russia**

Kuban State Medical University with affiliation Territorial Hospital #2: Igor Borisovich Zabolotskikh, Tatiana Sergeevna Musaeva

**Serbia**

UMC Zvezdara, Belgrad: Jovana Bojicic, Bojan Kovacevic

University Hospital Center "Dr Dragisa Misovic-Dedinje": Stevanovic Predrag, Dejan S Stojakov, Duska K Ignjatovic, Suzana C Bojic, Marina M Bobos, Irina B Nenadic, Milica S Zaric, Marko D Djuric, Vladimir R Djukic

CHC Bezanijska kosa, Belgrade: Marija Zdravkovic. Zoran Todorovic, Viseslav Popadic, Slobodan Klasnja

**Spain**

Hospital Universitario La Paz: Santiago Y. Teruel, Belen C. Martin

**Saudi Arabia**

King Saud University: Mohammed A Almazyad, Mohammed I Alarifi, Jara M Macarambon, Ahmad Abdullah Bukhari, Hussain A. Albahrani, Kazi N Asfina, Kaltham M Aldossary

King Fahad Armed Forces Hospital: Razan K Alamoudi, Hassan M. AlSharif, Sarah A. Almazwaghi, Mohammed S Elsakran, Mohamed A Aid, Mouaz A Darwich, Omnia M Hagag, Salah A Ali, Alona rocacorba, Kathrine Supeña, Efren Ray Juane, Jenalyn Medina, Jowany Baduria

King Faisal Specialist Hospital & Research Centre - Riyadh: Marwa Ridha Amer, Mohammed Abdullah Bawazeer, Talal I. Dahhan, Eiad Kseibi, Abid Shahzad Butt, Syed Moazzum Khurshid, Muath Rabee, Mohammed Abujazar, Razan Alghunaim, Maal Abualkhair, Abeer Turki AlFirm, Eiad Kseibi, Syed Moazzum Khurshid, Muath Rabee, Mohammed Abujazar, Razan Alghunaim

**Turkey**

Marmara University: Uluhan Sili, Huseyin Bilgin, Pinar Ay

**United States**

Baptist Health South Florida: Donna Lee Armaignac, Don Parris, Maria Pilar Zuniga, Ilea Vargas, Viviana Boronat, Anneka Hutton, Navneet Kaur, Prashank Neupane, Nohemi Sadule-Rios, Lourdes M. Rojas, Aashish Neupane, Priscilla Rivera, Carlos Valle Carlos, Gregory Vincent

Mayo Clinic Rochester: Rahul Kashyap, Juan Pablo Domecq, Ognjen Gajic, Vikas Bansal, Aysun Tekin, Amos Lal, John C. O'Horo, Neha N. Deo, Mayank Sharma, Shahraz Qamar, Romil Singh, Diana J. Valencia Morales

Mayo Clinic Arizona: Rodrigo Cartin-Ceba, Ayan Sen, Fahimeh Talaei

Atrium Health Navicent: Amy B. Christie, Dennis W. Ashley, Rajani Adiga

Mayo Clinic, Florida: Devang Sanghavi, Pramod Guru, Pablo Moreno Franco, Karthik Gnanapandithan, Hollie Saunders, Zachary Fleissner, Juan Garcia , Alejandra Yu Lee Mateus, Siva Naga Yarrarapu, Nirmaljot Kaur, Abhisekh Giri, Mohammed Mustafa Hasan, Ashrita Donepudi

Wake Forest University School of Medicine; Wake Forest Baptist Health Network: Ashish K. Khanna, Lynnette Harris, Bruce Cusson, Brandon Reeves, Jessica Fanelli, Nataya Disher, Anusha Samant, Chritian DeGroot, Evan Youshock, R. Miller Ligon, Katherine McCartney, Julio Garcia, Chidi Iloabachie, Kelsey Flores, David VanEenenaam, Lauren Sands, Samuel Robinson, Nia Sweatt, Jacob Fowler, Madeline Fram, Easton Howard, Kathleen Johnson, Imoh Udoh, Lillian Nosow, Lucy Winesett Howard, Quan Duc Minh Pham, Aman Irfanullah, Tiffany Ong, Chukwunyelu Henry Enwezor, Hannah Dabagian, Rafael Mendoza

Mayo Clinic, Eau Claire: Abigail T. La Nou, Marija Bogojevic, Simon Zec

University of Cincinnati: Dina Gomaa B.S., Michael Goodman, Devin Wakefield, Anthony Spuzzillo, John O. Shinn II

Mayo Clinic, Mankato: Syed Anjum Khan, Nitesh Kumar Jain, Thoyaja Koritala, Anwar Khedr, Hisham Mushtaq, Abbas Jama

St. Joseph Mercy Ann Arbor, Ann Arbor: Harry L. Anderson, III, Dixy Rajkumar, Ali Abunayla, Jerrilyn Heiter

Beth Israel Deaconess Medical Center: Valerie Banner Goodspeed, Lauren Kelly, Krystal Capers, Melisa Joseph, Lauryn Tsai.

Detar Family Medicine residency: Sidney Ontai, Brian Contreras, MD, Uzoma Obinwanko, Nneka Amamasi, Amir Sharafi

Howard University Hospital: Norma Smalls

The University of Tennessee Medical Center: Caleb Darby, Kristy Page, Amanda Brown, Jessie McAbee

Banner University Medical Center-Tucson: Jarrod M Mosier, Karen Lutrick, Beth Salvagio Campbell, Cathleen Wilson, Patrick Rivers, Jonathan Brinks, Mokenge Ndiva Mongoh, Boris Gilson

Cox Medical Center Springfield: Steven K. Daugherty, Sam Atkinson, Kelly Shrimpton

St. Joseph's Candler Health System: Howard A. Zaren, Stephanie J. Smith, Grant C. Lewis, Lauren Seames, Cheryl Farlow, Judy Miller, Gloria Broadstreet

Mercy Hospital, Saint Louis: Chakradhar Venkata, Miriam Engemann, Annamarie Mantese

Ridgecrest Regional Hospital: Victoria Schauf, Chris Wall

The Children's Hospital at OU Medicine: Neha Gupta, Tracy L Jones, Shonda C Ayers, Amy B Harrell, Brent R Brown, OU Medical Center: Neha Gupta, Brent R Brown, Tracy L Jones, Kassidy Malone, Lauren A Sinko, Amy B Harrell, Shonda C Ayers, Lisa M Settle, Taylor J Sears

Boston University School of Medicine, Boston, MA: Allan J. Walkey, Sushrut S. Waikar, Michael A. Garcia, Mia Colona, Zoe Kibbelaar, Michael Leong, Daniel Wallman, Kanupriya Soni, Jennifer Maccarone, Joshua Gilman, Ycar Devis, Joseph Chung, Munizay Paracha, David N. Lumelsky, Madeline DiLorenzo, Najla Abdurrahman, Shelsey Johnson

Stamford Health: Michael A. Bernstein, Ian K. Goff, Matthew Naftilan, Amal Mathew, Deborah Williams, Sue Murdock, RN, Maryanne Ducey, Kerianne Nelson

MacNeal Hospital Loyola Medicine: Christine C. Junia, Robert Lichtenberg, Hasrat Sidhu, Diana Espinoza, Shelden Rodrigues, Maria Jose Zabala, Daniela Goyes, Ammu Susheela, Buddhi Hatharaliyadda, Naveen Rameshkumar, Amulya Kasireddy, Genessis Maldonado, Lisseth Beltran, Akshata Chaugule, Hassan Khan

Lakes Region General Hospital: Michael Smith, William Snow, Riley Liptak, Hannah Durant, Valerie Pendleton, Alay Nanavati, Risa Mrozowsk, Erica Doubleday

Parkview Health System, Fort Wayne: Roger Scott Stienecker, Andre G. Melendez, Tressa A. Brunner, Sue M Budzon, Jessica L. Heffernan, Janelle M. Souder, Tracy L. Miller, Andrea G. Maisonneuve

Ascension/St. Thomas Research Institute West Campus: Stephen Capizzi, Bethany Alicie, Martha Green, Lori Crockarell, Amelia Drennan, Kathleen Dubuque, Tonya Fambrough, Nikole Gasaway, Briana Krantz, Peiman Nebi, Jan Orga, Margaret Serfass, Alina Simion, Kimberly Warren, Cassie Wheeler, CJ Woolman

Mercy Gilbert Medical Center: Jessica Timmer, Kimberly Welker

Tulane University Medical Center and University Medical Center New Orleans: Joshua L. Denson, A. Scott Gillet, Margo Brown, Rachael Stevens, Andrew Wetherbie, Kevin Tea, Mathew Moore

University of Vermont Larner College of Medicine: Renee D. Stapleton, Anne E. Dixon, Olivia Johnson, Sara S. Ardren, Stephanie Burns, Anna Raymond, Erika Gonyaw, Kevin Hodgdon, Chloe Housenger, Benjamin Lin, Karen McQuesten, Heidi Pecott-Grimm, Julie Sweet, Sebastian Ventrone

University of Kansas Medical Center: Usman Nazir, Garrett Rampon, Jake Riggle, Nathan Dismang

Baylor Scott & White Health: Valerie C. Danesh, Gueorgui Dubrocq, Amber L. Davis, Marissa J Hammers, ill M. McGahey, Amanda C. Farris, Elisa Priest, Robyn Korsmo, Lorie Fares, Kathy Skiles, Susan M. Shor, Kenya Burns, Corrie A Dowell, Gabriela “Hope” Gonzales, Melody Flores, Lindsay Newman, Debora A Wilk, Jason Ettlinger, Jaccallene Bomar, Himani Darji, Alejandro Arroliga, Alejandro C Arroliga, Corrie A. Dowell, Gabriela Hope Conzales, Melody Flores, Lindsay Newman, Debora A. Wilk, Jason Ettlinger, Himani Darji, Jaccallene Bomar

University of Michigan Health System: Pauline Park, Andrew Admon, Sinan Hanna, Rishi Chanderraj, Maria Pliakas, Ann Wolski, Jennifer Cirino

Samaritan Health Services: Brian L. Delmonaco, Anthony Franklin, Mitchell Heath

Sarasota Memorial Hospital: Antonia L. Vilella, Sara B. Kutner, Kacie Clark, Danielle Moore

OSF Saint Francis Medical Center: Bhagat S. Aulakh, Sandeep Tripathi, Jennifer A. Bandy, Lisa M. Kreps, Dawn R. Bollinger

University Clinical Hospital, Mostar, Bosnia and Herzegovina: Dragana Markotić, Ivana Bošnjak

University of Florida Health Shands Hospital: Azra Bihorac, Tezcan Ozrazgat Baslanti, George Omalay, Haleh Hashemighouchani, Julie S. Cupka, Matthew M Ruppert

Baylor College of Medicine, Baylor St. Lukes Medical Center: Christopher M Howard, Cameron McBride, Jocelyn Abraham, Orlando Garner, Katherine Richards, Keegan Collins, Preethi Antony, Sindhu Mathew

Thomas Jefferson University Hospital: Katherine A. Belden, Michael Baram, Devin M. Weber, Rosalie DePaola, Yuwei Xia, Hudson Carter, Aaron Tolley, Mary Ferranti

Augusta Health: Andrew S. Moyer, George M. Verghese

Washington University School of Medicine and Barnes-Jewish Hospital: Patrick G. Lyons, Andrew P Michelson, Sara S. Haluf, Lauren M. Lynch, Nguyet M. Nguyen, Aaron Steinberg

University Medical Center (University Medical Center of Southern Nevada Las Vegas): Rajany V. Dy, Alfredo Iardino, Jill Sharma, Richard Czieki, Julia Christopher, Ryan Lacey, Marwan Mashina,, Kushal Patel

Wyoming Medical Center: Vishwanath Pattan, Jessica Papke, Ismail Jimada, Nida Mhid, Samuel Chakola

University of Miami Miller School of Medicine: Roger A. Alvarez, Amarilys Alarcon-Calderon, Marie Anne Sosa, Sunita K. Mahabir, Mausam J. Patel

University of Iowa Carver College of Medicine: Patrick W. McGonagill, Colette Galet, Janice Hubbard, David Wang, Lauren Allan, Aditya Badheka, Madhuradhar Chegondi

Medical College of Wisconsin: Rahul S Nanchal, Paul A Bergl, Jennifer L Peterson

M Health-Fairview, University of Minnesota: Ronald A. Reilkoff, Julia A. Heneghan, Sarah Eichen, Lexie Goertzen, Scott Rajala, Ghislaine Feussom, Ben Tang

University of Missouri, Columbia: Dima Dandachi, Hariharan Regunath, Maraya N. Camazine, Grant. E. Geiger, Abdoulie O. Njai, Baraa M. Saad

Saint Alphonsus Regional Medical Center: Kerry P. J. Pulver, Jennifer Yehle, Alicia Weeks, Terra Inman

Aultman Hospital: Moldovan Sabov, Fatema Zaidi, Fiona Tissavirasingham, Dhatri Malipeddi

Mercy Hospital and Medical Center, Chicago: Travis Yamanaka, Nicholas A. Barreras, Michael Markos, Anita Fareeduddin, Rohan Mehta

UNC Medical Center: Benjamin J Sines, Thomas J Bice

Yale New Haven Health New Haven: Kevin Sheth, Abdalla Ammar, Mahmoud Ammar, Victor Torres Lopez , Charles Dela Cruz, Akhil Khosla, Samir Gautam

Ascension St.Vincent Hospital: Anmol Kharbanda, Sunil Jhajhria, Zachary Fyffe

Chambersburg Hospital: Raghavendra Tirupathi, Alymer Tang, Arshad Safi, Cindy Green, Jackie Newell, Naga Ramani, Bhavani Harika Ganti

University of Arkansas for Medical Sciences: Nikhil K. Meena, Jose D. Caceres, Nikhil K Meena, Sarenthia M. Epps, Harmeen Goraya, Kelsey R. Besett, MD, Ryan James, Lana Y. Abusalem, Akash K. Patel, Lana S Hasan

AnMed Health: Abhijit A Raval, Andrea Franks

Stanford Hospital and Clinics: Jason Block, James Mitchel, Connor G O'Brien, Sylvan Cox

Millard Fillmore Suburban Hospital: Anna Eschler, Mary Hejna, Emily Lewandowski, Kristen Kusmierski, Clare Martin

JPS Health Network: Steven Q. Davis, Valentina Jovic, Valentina Jovic, Max Masuda, Amanda Hayes

George Washington University: David P. Yamane, Ivy Benjenk, Nivedita Prasanna

St. Agnes Hospital: Anthony Martinez, Micheal Allison, Aniket Mittal, Rafael Ruiz, Aleta Skaanland, Robert Ross

Loyola University Medical Center: Yuk Ming Liu, Sarah Zavala, Sarah Zavala, Esther Shim

Montefiore Medical Center The Bronx: Jen-Ting Chen, Aluko Hope, Zoe Tsagaris, Elise Ruen, Aram Hambardzumyan
